# Toll-like receptor-4 differentially mediates intestinal and extra-intestinal immune responses upon multi-drug resistant *Pseudomonas aeruginosa* association of IL10^−/−^ mice with chronic colitis

**DOI:** 10.1186/s13099-017-0211-z

**Published:** 2017-11-07

**Authors:** Anne Grunau, Ulrike Escher, Anja A. Kühl, Stefan Bereswill, Markus M. Heimesaat

**Affiliations:** 1 Department of Microbiology and Hygiene, Charité-Universitätsmedizin Berlin, corporate member of Freie Universität Berlin, Humboldt-Universität zu Berlin, Berlin, Germany; 2 Department of Medicine I for Gastroenterology, Infectious Diseases and Rheumatology/Research Center ImmunoSciences (RCIS), Charité-Universitätsmedizin Berlin, corporate member of Freie Universität Berlin, Humboldt-Universität zu Berlin, Berlin, Germany; 3 Berlin Institute of Health, Berlin, Germany; 4 CC5, Department of Microbiology and Hygiene, Charité-Universitätsmedizin Berlin, Campus Benjamin Franklin, FEM, Garystr. 5, 14195 Berlin, Germany

**Keywords:** *Pseudomonas aeruginosa*, Multi-drug resistant Gram-negative bacteria, Colonization resistance, Susceptibility to infection, Intestinal microbiota shifts, Chronic IL10^−/−^ colitis, Pro-inflammatory immune responses, Extra-intestinal sequelae of infection, Bacterial translocation, Toll-like receptor-4, Lipopolysaccharide

## Abstract

**Background:**

Infections with multi-drug resistant (MDR) Gram-negative bacteria including *Pseudomonas aeruginosa* (PA) have become a serious threat particularly in hospitalized patients with immunopathological co-morbidities. The well-balanced interplay between immune cells, pattern recognition receptors such as Toll-like receptor (TLR)-4 sensing lipopolysaccharide from Gram-negative bacteria including PA, and evolving pathways is crucial to prevent the host from invading (opportunistic) pathogens. Information regarding the molecular mechanisms underlying the interactions between intestinal carriage of MDR PA and host immunity during chronic large intestinal inflammation is scarce, however.

**Methods and results:**

We therefore perorally challenged conventionally colonized TLR4-deficient IL10^−/−^ mice and IL10^−/−^ counterparts displaying comparably severe chronic colitis with a clinical MDR PA strain. PA could more sufficiently establish in the intestinal tract of TLR4-deficient IL10^−/−^ mice until day 14 postinfection (p.i.), whereas within 48 h the majority of IL10^−/−^ mice had already expelled the opportunistic pathogen from their guts. Intestinal colonization properties of PA in TLR4-deficient IL10^−/−^ mice were associated with distinct genotype-dependent differences in gut microbiota compositions before challenge given that TLR4-deficient IL10^−/−^ mice harbored more fecal enterobacteria and enterococci, but lower *Clostridium/Eubacterium* burdens. At day 14 p.i., PA-induced increases in colonic immune cells such as macrophages, monocytes and T-lymphocytes could be observed in TLR4-deficient IL10^−/−^ mice, but not IL10^−/−^ counterparts, that were accompanied by a more distinct secretion of IFN-γ in the colon and TNF in the mesenteric lymph nodes (MLN) of the former as compared to the latter. Conversely, splenic TNF levels were lower in TLR4-deficient IL10^−/−^ mice as compared to IL10^−/−^ controls at day 14 p.i. Interestingly, more pronounced apoptotic responses could be assessed in colonic epithelia of PA-challenged IL10^−/−^ mice only. This was paralleled by enhanced pro-inflammatory cytokine secretion not only in the intestines, but also in extra-intestinal compartments of IL10^−/−^ mice as indicated by increased concentrations of nitric oxide in the colon, IFN-γ in the MLN and IL-12p70 in the spleen at day 14 p.i.

**Conclusions:**

Under chronic intestinal inflammatory conditions including IL10^−/−^ colitis MDR PA-association results in well-orchestrated TLR4-dependent immune responses both in intestinal and extra-intestinal compartments. Further studies should unravel the underlying molecular mechanisms in more detail.

**Electronic supplementary material:**

The online version of this article (10.1186/s13099-017-0211-z) contains supplementary material, which is available to authorized users.

## Background

*Pseudomonas aeruginosa* (PA) are strictly aerobic Gram-negative bacteria that can be ubiquitously found, particularly in moist environments [1]. A polar flagellum and type 4 pili enable bacterial motility and adherence to surfaces, but can also initiate inflammatory responses. Alginate secretion and biofilm formation, quorum sensing, proteases and an elaborated type 3 secretion system further contribute to a diverse arsenal of virulence factors facilitating immune escape and establishment in the host ecosystem [2, 3]. The well-balanced interplay between immune cells, pattern recognition receptors such as Toll-like receptors (TLRs) and evolving pathways is crucial to prevent the host from invading (opportunistic) pathogens. In this context TLR4 senses PA lipopolysaccharide (LPS), which leads to activation of the NFκB signaling pathway and subsequent induction of pro-inflammatory cytokine and chemokine secretion with potentially fatal outcome [4, 5]. PA is regarded as opportunistic pathogen and constitutes one of the most prominent causes of acute nosocomial infections, preferably affecting immune-compromised individuals with severe co-morbidities and patients admitted to the intensive care unit (ICU) [6, 7]. PA-related infections include ventilator-associated pneumonia, infections of surgical sites, burn wounds, and of the urinary tract, associated with mortality rates of > 30% depending both on the immune status of the host and the antimicrobial resistance of the bacterial strain [6]. PA is further known to drive chronic respiratory infections in patients suffering from cystic fibrosis, bronchiectasis or chronic obstructive pulmonary disease [7–9]. The alarming scenario of globally emerging infections with strains that are resistant to several antimicrobial classes due to β-lactamases, carbapenemases and 16S rRNA methylases [7, 9, 10] has prompted the World Health Organization (WHO) to rate multi-drug resistant (MDR) Gram-negative species including PA as serious threats for human health [11]. Whereas in the hospital setting respiratory equipment, water bottles, and sinks constitute typical environmental reservoirs [12], the human gastrointestinal tract might be considered as internal source for PA infection [13, 14]. Even though not considered as being part of the human commensal intestinal microbiota, intestinal PA colonization is a prerequisite of infection later on, given that rectally colonized individuals harbored a 15 times higher risk for development of a PA infection when admitted to an ICU [15]. Of note, intestinal PA colonization rates of patients have been shown to increase during hospital stay presumably due to exposure to contaminated surfaces on wards or colonized patients via cross-contamination [13]. Particularly antibiotic treatment facilitated the establishment of PA in the patients’ gastrointestinal ecosystem, given that antimicrobials also affected the complex intestinal microbiota composition that usually prevents the host from invading (opportunistic) pathogens [7, 16]. Recent surveys revealed that intestinal PA detection rates were higher in patients with intestinal inflammatory morbidities including irritable bowel syndrome [17] or inflammatory bowel disease (IBD) such as ulcerative colitis [18]. Whereas the pathogenic potential of PA is well known given its diverse arsenal of virulence factors, our knowledge regarding the interplay of PA, commensal intestinal microbiota and host immunity, particularly under conditions of intestinal inflammation, is scarce, however. This prompted us to study gastrointestinal colonization properties and potential TLR4 dependent intestinal as well as extra-intestinal immune responses upon MDR PA challenge under chronic intestinal inflammatory conditions applying the chronic IL10^−/−^ colitis mouse model. Conventionally colonized but not germfree or secondary abiotic IL10^−/−^ mice develop chronic large intestinal inflammation due to antigenic stimuli derived from their commensal gut microbiota with progressive aging and are considered a valuable mouse model for human intestinal inflammation such as IBD affection the colon [19].

## Methods

### Ethical statement

All animal experiments were conducted according to the European Guidelines for animal welfare (2010/63/EU) with approval of the commission for animal experiments headed by the “Landesamt für Gesundheit und Soziales” (LaGeSo, Berlin; Registration Numbers G0097/12 and G0039/15). Animal welfare was monitored twice daily by assessment of clinical conditions of mice.

### Mice and *P. aeruginosa* infection

Female and male IL10^−/−^ mice as well as TLR4 deficient IL10^−/−^ mice (TLR4^−/−^ × IL10^−/−^), all in C57BL/10ScSn background, were reared and maintained within the same specific pathogen free (SPF) unit of the Forschungseinrichtungen für Experimentelle Medizin (FEM, Charité-University Medicine Berlin). Sex matched, between 11 and 14 months old mice of either genotype were perorally challenged with 10^9^ colony forming units (CFU) of a MDR *P. aeruginosa* strain by gavage in a total volume of 0.3 mL phosphate buffered saline (PBS; Ginco, life technologies, UK) as reported earlier [20]. The *P. aeruginosa* isolate was cultured from respiratory material of a patient with nosocomial pneumonia and kindly provided by Prof. Dr. Bastian Opitz (Charité-University Medicine, Berlin, Germany). Of note, the bacterial strain was tested resistant against piperacillin/tazobactam, ceftazidime, cefepim ± cefoxitin, imipenem, ciprofloxacin, gentamycin, tobramycin, amikacin, trimethoprim/sulfamethoxazole and aztreonam (according to EUCAST interpretation guidelines) and displayed antimicrobial sensitivity to fosfomycin and colistin only [20].

### Cultural analysis of *P. aeruginosa*

For quantitative assessment of *P. aeruginosa* colonization properties, fecal and luminal samples derived from distinct parts of the gastrointestinal tract such as stomach, duodenum, ileum and colon were homogenized in sterile PBS. Serial dilutions were streaked onto Columbia agar supplemented with 5% sheep blood and Cetrimid agar (both Oxoid, Germany) and incubated in an aerobic atmosphere at 37 °C for at least 48 h as described previously [20].

### Clinical conditions

To assess clinical signs upon PA challenge on a daily basis, a standardized cumulative clinical score (maximum 12 points), addressing the occurrence of blood in feces (0: no blood; 2: microscopic detection of blood by the Guajac method using Haemoccult, Beckman Coulter/PCD, Germany; 4: macroscopic blood visible), diarrhea (0: formed feces; 2: pasty feces; 4: liquid feces), and the clinical aspect (0: normal; 2: ruffled fur, less locomotion; 4: isolation, severely compromised locomotion, pre-final condition) was used as described earlier [21, 22].

### Sampling procedures and histopathology

Mice were sacrificed 14 days postinfection (p.i.) by isoflurane treatment (Abbott, Germany). Cardiac blood and tissue samples from spleen, liver, kidney, lung, mesenteric lymph nodes (MLN), ileum and colon were removed under sterile conditions. Intestinal samples were collected from each mouse in parallel for microbiological, immunohistochemical and histopathological analyses. Histopathological changes were determined in samples derived from the colon that were immediately fixed in 5% formalin and embedded in paraffin. Sections (5 μm) were stained with hematoxylin and eosin (H&E), examined by light microscopy (magnification 100× and 400×) and histopathological changes quantitatively assessed applying an established histopathological scoring system (maximum 4 points) as described previously [23].

### Immunohistochemistry

Colonic paraffin sections (5 μm) were subjected to in situ immunohistochemical analysis as described earlier [22, 24]. In brief, primary antibodies against cleaved caspase-3 (Asp175, Cell Signaling, Beverly, MA, USA, 1:200), Ki67 (TEC3, Dako, Glostrup, Denmark, 1:100), F4/80 (# 14-4801, clone BM8, eBioscience, 1:50), CD3 (#N1580, Dako, 1:10), FOXP3 (FJK-16 s, eBioscience, San Diego, CA, USA, 1:100) and B220 (eBioscience, 1:200) were used to detect apoptotic cells, proliferating cells, macrophages/monocytes, T lymphocytes, regulatory T cells (Treg) and B lymphocytes, respectively. The average numbers of positively stained cells within at least six high power fields (HPF, 0.287 mm^2^; 400× magnification) were determined by an independent blinded investigator.

### Cultural survey of intestinal microbiota

For comprehensive quantitative survey of the intestinal microbiota composition, colonic luminal contents were homogenized in sterile PBS and analyzed in serial dilutions on respective solid media as described earlier [25–27]. Bacteria were grown at 37 °C for at least 2 and 3 days under aerobic, microaerobic and anaerobic conditions. Following subcultivation of bacteria with distinct colony morphotypes, respective species were identified by biochemical analysis and 16S rRNA sequencing [25–27]. The detection limit of viable bacteria was approximately 100 CFU/g.

### Cytokine detection

Ex vivo biopsies (approximately 1 cm^2^) derived from colon (cut longitudinally and washed in PBS), MLN and spleen were placed in 24-flat-bottom well culture plates (Falcon, Germany) containing 500 mL serum-free RPMI 1640 medium (Gibco, life technologies) supplemented with penicillin (100 U/mL, Biochrom, Germany) and streptomycin (100 µg/mL; Biochrom). After 18 h at 37 °C, culture supernatants were tested for IFN-γ, TNF and IL-12p70 by the Mouse Inflammation Cytometric Bead Assay (CBA; BD Bioscience) on a BD FACSCanto II flow cytometer (BD Bioscience). Nitric oxide (NO) was determined by the Griess reaction as described earlier [25]. Obtained cytokine levels were normalized to mg protein.

### Statistical analysis

Mean values, medians, and levels of significance as assessed by Mann–Whitney-U Test and Wilcoxon Test were determined using GraphPad Prism Software v5 (La Jolla, CA, USA). Two-sided probability (p) values ≤ 0.05 were considered significant. Experiments were repeated twice.

## Results

### Intestinal colonization properties of multi-drug resistant *P. aeruginosa* following peroral association of TLR4 deficient IL10^−/−^ mice

**Fig. 1:**
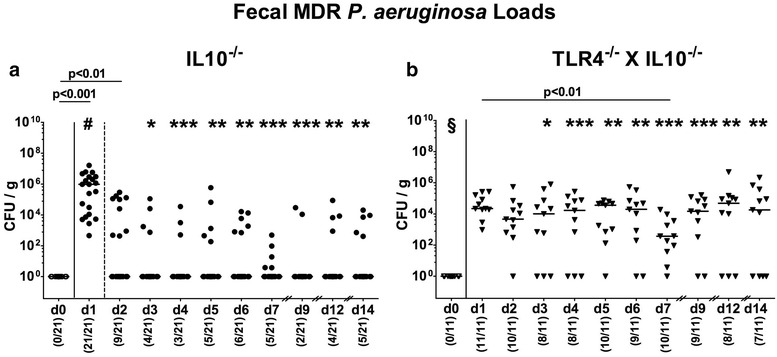
Intestinal colonization properties of multi-drug resistant *P. aeruginosa* following peroral association of IL10^−/−^ mice lacking TLR4. Conventionally colonized **a** IL10 deficient (IL10^−/−^; closed circles) and **b** TLR4 deficient IL10^−/−^ mice (TLR4^−/−^ × IL10^−/−^; closed triangles) were perorally challenged with a clinical multi-drug resistant *P. aeruginosa* strain at day (d) 0. Intestinal colonization densities were determined in fecal samples until d14 postinfection by culture and expressed as colony forming units per gram (CFU/g). Numbers of mice harboring *P. aeruginosa* out of the total number of analyzed mice (in parentheses), medians (black bars) and significance levels (p values) determined by Mann–Whitney U test and Wilcoxon Test are indicated. Asterisks illustrate significant differences between genotypes at defined time points (*p < 0.05; **p < 0.01; ***p < 0.001); ^#^p < 0.001 (d1 versus all other time points); ^§^p < 0.001 (d0 versus all other time points). p values in numbers indicate differences between mice of identical genotype at distinct time points as shown by the horizontal bar underneath. Data shown were pooled from at least three independent experiments

**Fig. 2:**
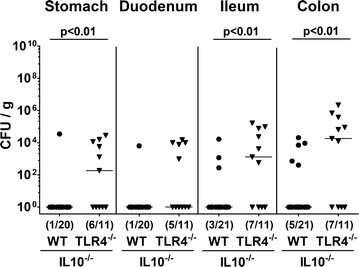
Multi-drug resistant *P. aeruginosa* loads in the gastrointestinal tract following peroral association of IL10^−/−^ mice lacking TLR4. Conventionally colonized IL10 deficient (WT IL10^−/−^; closed circles) and TLR4 deficient IL10^−/−^ mice (TLR4^−/−^ × IL10^−/−^; closed triangles) were perorally challenged with a clinical multi-drug resistant *P. aeruginosa* strain at day (d) 0. At necropsy (i.e. d14 postinfection), *P. aeruginosa* loads were quantitatively assessed in gastrointestinal luminal samples derived from stomach, duodenum, ileum und colon by culture and expressed as colony forming units per gram (CFU/g). Numbers of mice harboring *P. aeruginosa* out of the total number of analyzed mice (in parentheses), medians (black bars) and significance levels (p values) determined by Mann–Whitney U test are indicated. Data were pooled from at least three independent experiments

In order to address the impact of TLR4 in MDR PA colonization and subsequent intestinal as well as extra-intestinal immune responses in murine IL10^−/−^ colitis, we perorally challenged 11–14 months old conventionally colonized TLR4 deficient IL10^−/−^ mice and IL10^−/−^ counterparts with 10^9^ CFU of a clinical MDR PA isolate by gavage. Whereas 24 h later all IL10^−/−^ mice harbored the bacterial strain in their intestines with median loads of approximately 10^6^ CFU/g (p < 0.001 versus d0; Fig. 1a), fecal PA loads declined thereafter (p < 0.001 versus day 1 p.i.; Fig. 1a). Of note, as early as 48 h p.i., more than 50% of mice had already expelled the opportunistic pathogen from their intestinal tract, whereas later on, PA could be isolated from fecal samples in single cases only (Fig. 1a). In TLR4 deficient IL10^−/−^ mice, however, fecal PA loads were significantly higher as compared to IL10^−/−^ counterparts from day 3 p.i. until the end of the observation period (p < 0.05–0.001; Fig. 1b). In addition, only 23.8% of IL10^−/−^ mice harbored PA in their intestines at day 14 p.i., whereas 63.6% of fecal samples derived from TLR4 deficient IL10^−/−^ mice were PA-positive (Fig. 1). At necropsy, detection rates as well as bacterial burdens were higher in stomach, ileum and colon of TLR4 deficient IL10^−/−^ mice as compared to IL10^−/−^ controls (p < 0.01; Fig. 2). Hence, MDR PA could more sufficiently establish within the gastrointestinal tract of TLR4 deficient IL10^−/−^ mice.

### Intestinal microbiota composition before peroral association of TLR4 deficient IL10^−/−^ mice with multi-drug resistant *P. aeruginosa*

**Fig. 3:**
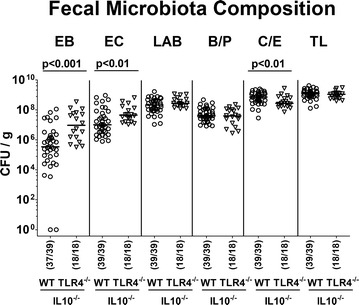
Intestinal microbiota composition before peroral association of IL10^−/−^ mice lacking TLR4 with multi-drug resistant *P. aeruginosa.* Immediately before peroral challenge with a clinical multi-drug resistant *P. aeruginosa* strain at day (d) 0, a comprehensive survey of the intestinal microbiota composition was performed on fecal samples derived from conventionally colonized IL10 deficient (WT IL10^−/−^; open circles) and TLR4 deficient IL10^−/−^ mice (TLR4^−/−^ × IL10^−/−^; open triangles), and the following intestinal commensal bacteria were assessed by culture (expressed as colony forming units per gram, CFU/g): Enterobacteria (EB), enterococci (EC), lactic acid bacteria (LAB), *Bacteroides/Prevotella* spp. (B/P), *Clostridium/Eubacterium* spp. (C/E) and total bacterial loads (TL). Numbers of mice harboring the respective commensal intestinal bacteria out of the total number of analyzed mice (in parentheses), medians (black bars) and significance levels (p values) determined by Mann–Whitney U test are indicated. Data were pooled from at least three independent experiments

Given that the intestinal microbiota composition constitutes the physiological colonization resistance preventing the host from (opportunistic) pathogenic infection [27], we next performed a comprehensive survey of the commensal intestinal microbiota composition in mice of either genotype by cultural analyses of fecal samples that had been taken immediately before PA challenge. Interestingly, TLR4 deficient IL10^−/−^ mice harbored up to two orders of magnitude higher numbers of commensal enterobacteria and enterococci in their intestinal tract at day 0 (p < 0.001 and p < 0.01, respectively; Fig. 3), whereas fecal obligate anaerobic *Clostridium/Eubacterium* spp. were lower as compared to IL10^−/−^ controls (p < 0.01, Fig. 3). Hence, TLR4-dependent differences in intestinal colonization properties of MDR PA were associated with genotype-dependent differences in intestinal microbiota compositions of IL10^−/−^ mice before peroral PA challenge and subsequently facilitated PA colonization in TLR4 deficient IL10^−/−^ mice.

### Macroscopic and microscopic sequelae in TLR4 deficient IL10^−/−^ mice following peroral association with multi-drug resistant *P. aeruginosa*

**Fig. 4:**
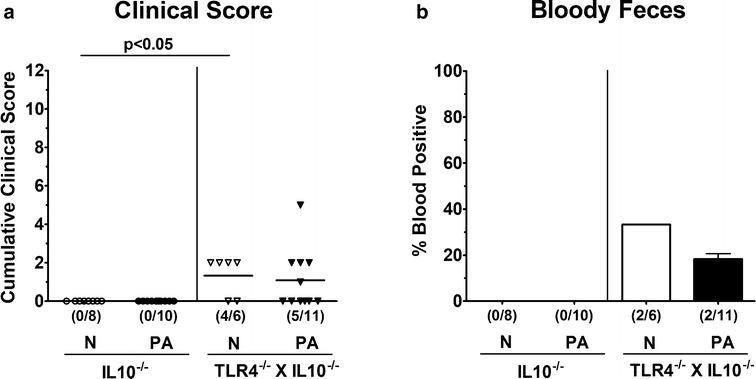
Clinical conditions in IL10^−/−^ mice lacking TLR4 following peroral association with multi-drug resistant *P. aeruginosa*. Conventionally colonized IL10 deficient (WT IL10^−/−^; closed circles) and TLR4 deficient IL10^−/−^ mice (TLR4^−/−^ × IL10^−/−^; closed triangles, black bar) were perorally challenged with a clinical multi-drug resistant *P. aeruginosa* (PA) strain at day (d) 0. **a** Clinical symptoms (applying a standardized clinical scoring system) and **b** fecal blood-positivity rates were surveyed at day 14 p.i. Naive (N) mice served as negative controls (white bar). Numbers of mice displaying clinical symptoms including blood-positive feces out of the total numbers of analyzed animals (in parentheses), median (black bars; **a**), blood-positivity rates ± standard deviations (in %; **b**) and significance levels (p values) determined by the Mann–Whitney U test are indicated. Data shown were pooled from three independent experiments

**Fig. 5:**
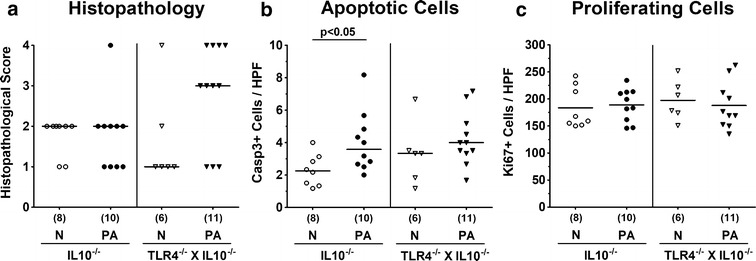
Histopathological mucosal changes, apoptotic and proliferating epithelial cells in large intestines of IL10^−/−^ mice lacking TLR4 following peroral association with multi-drug resistant *P. aeruginosa*. Conventionally colonized IL10 deficient (WT IL10^−/−^; closed circles) and TLR4 deficient IL10^−/−^ mice (TLR4^−/−^ × IL10^−/−^; closed triangles) were perorally challenged with a clinical multi-drug resistant *P. aeruginosa* (PA) strain at day (d) 0. Fourteen days thereafter **a** histopathological mucosal changes were assessed in hematoxylin and eosin stained colonic paraffin sections applying a standardized histopathological scoring system. In addition, the average numbers of colonic epithelial, **b** apoptotic (positive for caspase 3, Casp3) and **c** proliferating cells (positive for Ki67) were determined from six high power fields (HPF, 400× magnification) per animal in immunohistochemically stained large intestinal paraffin sections. Naive (N) mice served as negative controls (open symbols). Numbers of mice (in parentheses), median (black bars) and significance levels (p values) determined by the Mann–Whitney U test are indicated. Data shown were pooled from three independent experiments

We next assessed potential TLR4 dependent macroscopic and microscopic sequelae upon MDR PA colonization of IL10^−/−^ mice. Whereas in the basal state TLR4 deficient IL10^−/−^ mice displayed slightly higher clinical scores indicative for ruffled fur and/or microscopic abundance of fecal blood, but in single cases only (p < 0.05; Fig. 4a), clinical conditions were comparable at day 14 following PA colonization of mice of either genotype. Whereas neither in PA colonized nor in naive IL10^−/−^ mice fecal blood could be detected, this was the case in 33.3 and 18.2% of naïve and PA associated TLR4 deficient IL10^−/−^ mice, respectively (Fig. 4b). In line with their macroscopic aspects, mice of either genotype displayed chronic colitis of moderate but comparable severity (Fig. 5a) with mucosal and submucosal inflammatory infiltrates and dysplastic crypts that could be observed both before and 14 days after PA challenge (Additional file 1: Figure S1). Given that apoptosis is a suitable marker for grading of intestinal inflammation [28], we next quantitatively assessed colonic apoptosis applying in situ immunohistochemistry. At day 14 p.i., increased numbers of apoptotic cells could be observed in the large intestinal epithelia of IL10^−/−^ (p < 0.05), but not TLR4 deficient IL10^−/−^ mice (Fig. 5b). Of note, numbers of colonic Ki67+ cells indicative for cell proliferation and regeneration thereby counteracting potential cell damage were unchanged upon PA association of mice of either genotype (n.s.; Fig. 5c). Hence, apoptotic cell responses upon MDR PA colonization were more pronounced in large intestines of IL10^−/−^ mice.

### Colonic immune cell responses in TLR4 deficient IL10^−/−^ mice following peroral association with multi-drug resistant *P. aeruginosa*

**Fig. 6:**
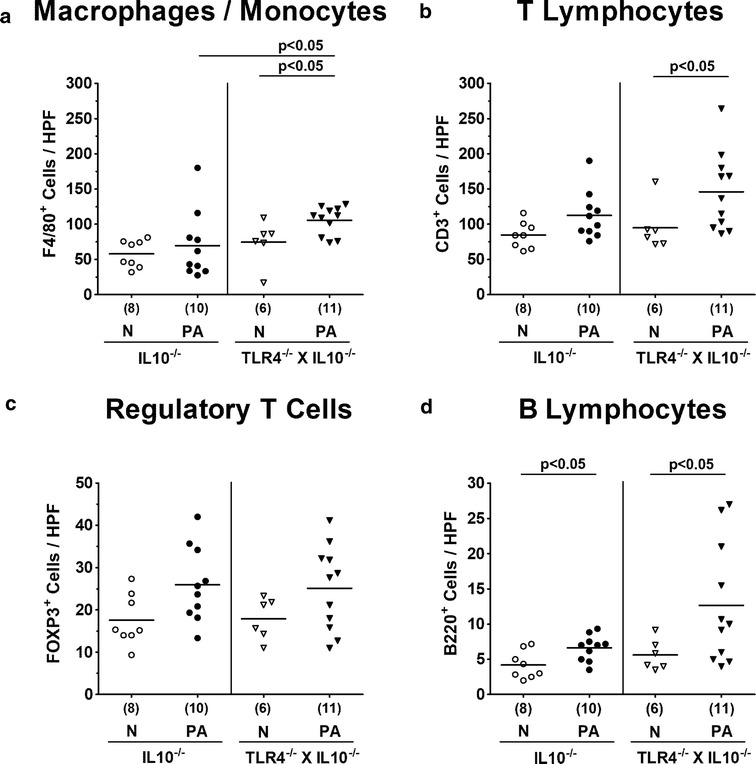
Colonic immune cell responses in IL10^−/−^ mice lacking TLR4 following peroral association with multi-drug resistant *P. aeruginosa*. Conventionally colonized IL10 deficient (WT IL10^−/−^; closed circles) and TLR4 deficient IL10^−/−^ mice (TLR4^−/−^ × IL10^−/−^; closed triangles) were perorally challenged with a clinical multi-drug resistant *P. aeruginosa* (PA) strain at day (d) 0. Fourteen days thereafter, the average numbers of colonic **a** macrophages and monocytes (positive for F4/80), **b** T lymphocytes (positive for CD3), **c** regulatory T cells (positive for FOXP3), and **d** B lymphocytes (positive for B220) were determined from six high power fields (HPF, 400× magnification) per animal in immunohistochemically stained large intestinal paraffin sections. Naive (N) mice served as negative controls (open symbols). Numbers of mice (in parentheses), medians (black bars) and significance levels (p values) determined by the Mann–Whitney U test are indicated. Data shown were pooled from three independent experiments

We next addressed whether PA colonization resulted in TLR4 dependent immune cell responses within the intestinal tract. We therefore quantitatively determined numbers of large intestinal innate (i.e. macrophages and monocytes) as well as adaptive (i.e. T lymphocytes, Treg and B lymphocytes) immune cell populations by in situ immunohistochemical staining of colonic paraffin sections with distinct antibodies. At day 14 p.i., increased numbers of macrophages and monocytes could be observed in the large intestinal mucosa and lamina propria of TLR4 deficient IL10^−/−^ mice (p < 0.05), but not IL10^−/−^ controls (Fig. 6a), which also held true for colonic T lymphocytes (p < 0.05; Fig. 6b). Whereas Treg numbers remained unchanged upon PA challenge (n.s.; Fig. 6c), B lymphocytes were elevated in large intestines at day 14 p.i., irrespective of the genotype of mice (p < 0.05; Fig. 6d). Hence, distinct innate as well as adaptive immune responses upon MDR PA colonization were more pronounced in large intestines of TLR4 deficient IL10^−/−^ mice.

### Intestinal and extra-intestinal cytokine responses in TLR4 deficient IL10^−/−^ mice following peroral association with multi-drug resistant *P. aeruginosa*

**Fig. 7:**
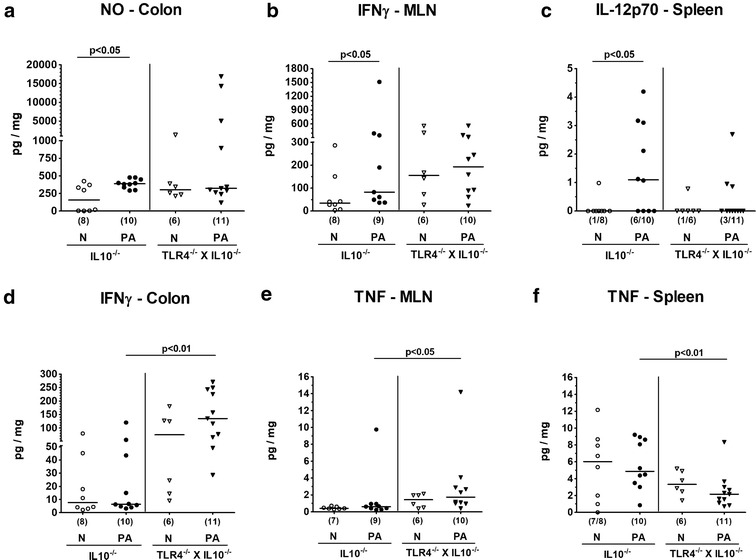
Intestinal and splenic cytokine responses in IL10^−/−^ mice lacking TLR4 following peroral association with multi-drug resistant *P. aeruginosa*. Conventionally colonized IL10 deficient (WT IL10^−/−^; closed circles) and TLR4 deficient IL10^−/−^ mice (TLR4^−/−^ × IL10^−/−^; closed triangles) were perorally challenged with a clinical multi-drug resistant *P. aeruginosa* (PA) strain at day (d) 0. Fourteen days thereafter, secretion of distinct pro-inflammatory mediators were measured in supernatants of ex vivo biopsies derived from colon (**a** nitric oxide (NO); **d** IFN-γ), mesenteric lymph nodes (MLN; **b** IFN-γ; **e** TNF) and spleen (**c** IL-12p70; **e** TNF). Naive (N) mice served as negative controls (open symbols). Numbers of mice (in parentheses), medians (black bars) and significance levels (p values) determined by the Mann–Whitney U test are indicated. Data shown were pooled from three independent experiments

We next assessed whether observed TLR4 dependent differences in colonic immune responses were associated with genotype dependent intestinal and even extra-intestinal cytokine secretion. More pronounced apoptotic responses in colonic epithelia that had been observed exclusively following PA challenge of IL10^−/−^ mice were paralleled by enhanced pro-inflammatory cytokine secretion not only in the intestinal tract but also in extra-intestinal compartments as indicated by increased concentrations of NO in the colon (p < 0.05; Fig. 7a), IFN-γ in the MLN (p < 0.05; Fig. 7b) and IL-12p70 in the spleen (p < 0.05; Fig. 7c) of IL10^−/−^ mice at day 14 p.i.

More prominent colonic abundances of macrophages, monocytes and T lymphocytes in PA colonized TLR4 deficient IL10^−/−^ mice, however, were accompanied by more distinct secretion of IFN-γ in colon (p < 0.01; Fig. 7d) and TNF in MLN (p < 0.05; Fig. 7e) of TLR4 deficient IL10^−/−^ mice as compared to IL10^−/−^ counterparts, whereas conversely, splenic TNF levels were lower in the former than the latter at day 14 p.i. (p < 0.01; Fig. 7f). Hence, distinct TLR4 dependent pro-inflammatory immune response scenarios following intestinal colonization with MDR PA are mirrored by distinct intestinal and even extra-intestinal (i.e. splenic) pro-inflammatory cytokine responses.

## Discussion

It is under current debate whether mere intestinal carriage of the opportunistic pathogen PA does have any immunopathological impact in health and disease [20, 29]. In a previous survey, we were able to show that following peroral challenge of clinically uncompromised microbiota-depleted mice with an identical clinical isolate, MDR PA was not only able to stably colonize the intestinal tract at high loads, but also to induce intestinal as well as even extra-intestinal (i.e. splenic) pro-inflammatory immune responses [29]. Hence, mere intestinal carriage of MDR Gram-negative opportunistic pathogens such as PA by an antibiotic-pretreated, but otherwise asymptomatic individual might result in immunopathological sequelae that may even be aggravated by other comorbidities (and vice versa). This prompted us to unravel the interplay of MDR PA with the commensal intestinal microbiota and host immunity during chronic inflammation of the large intestinal tract here. Following peroral challenge, approximately two-thirds of TLR4 deficient IL10^−/−^, but only up to 25% of IL10^−/−^ counterparts harbored MDR PA in their intestine 2 weeks following peroral challenge. Given that the intestinal microbiota composition determines host resistance against invading (opportunistic) pathogens [28], we surveyed the intestinal gut microbiota before peroral challenge of mice of either genotype. Cultural analyses revealed that naive, conventionally colonized TLR4 deficient IL10^−/−^ mice harbored up to two orders of magnitude higher fecal enterobacteria as compared to IL10^−/−^ controls. In our previous studies we were able to demonstrate that elevated intestinal loads of enterobacterial commensals such as *Escherichia coli* were able to override physiological colonization resistance and facilitated murine infection with the enteropathogen *Campylobacter jejuni* [30–32]. One also needs to take into consideration that intestinal inflammation per se might facilitate pathogenic colonization [20, 25, 32, 33]. Before PA challenge histopathological severities of chronic colitis were comparable in either mice, however, and were not further aggravated upon PA challenge until the end of the study. The TLR4 dependent colonization properties of MDR PA observed here are supported by our very recent survey where we could show that healthy microbiota-depleted TLR4 deficient IL10^−/−^ mice harbored higher PA burdens in their gastrointestinal tract as compared to microbiota-depleted IL10^−/−^ counterparts [34]. The PA induced intestinal and splenic pro-inflammatory immune responses do not follow a clear-cut pattern when comparing TLR4 deficient IL10^−/−^ mice and IL10^−/−^ controls, however. On one hand, besides higher intestinal opportunistic pathogenic loads, only MDR PA colonized TLR4 deficient IL10^−/−^ mice displayed increased numbers of innate as well as of adaptive immune cell populations such as macrophages and monocytes as well as of T lymphocytes in their large intestinal mucosa and lamina propria at day 14 p.i. These increases in immune cells were accompanied by more pronounced intestinal secretion of pro-inflammatory cytokines such as IFN-γ and TNF in colon and MLN, respectively. Of note, splenic TNF concentrations were lower in TLR4 deficient IL10^−/−^ mice as compared to IL10^−/−^ counterparts at day 14 p.i., which might be explained by more pronounced recruitment of leukocytes to the intestinal tract. One might argue that the more distinct immune responses observed in TLR4 deficient IL10^−/−^ mice were merely due to better gastrointestinal colonization properties of MDR PA as compared to IL10^−/−^ control mice. One needs to take into consideration, however, that an (opportunistic) pathogen does not necessarily need to be permanently abundant within the gastrointestinal tract in order to induce immunopathological responses [22, 35–37]. It is rather the initial hit by the bacterial stimulus tipping the balance towards pro-inflammatory immune responses during (opportunistic) pathogen–host interactions [35]. In fact, despite only sporadic colonization, IL10^−/−^, but not TLR4 deficient IL10^−/−^ mice, on the other side, displayed increased numbers of apoptotic colonic epithelial cells that were paralleled by increased secretion of NO and IFN-γ in colon and MLN, respectively, whereas elevated IL-12p70 levels could be measured in extra-intestinal compartments like the spleen. In support, our very recent study revealed that TLR4 mediated PA induced inflammatory responses in otherwise healthy secondary abiotic IL10^−/−^ mice without colitis [34]. Lipid A constitutes a core moiety of LPS derived from Gram-negative bacteria including *Pseudomonas* and has been shown to activate NFκB signaling via TLR4 leading to increased pro-inflammatory cytokine expression [38]. In turn, innate immune cells are recruited to the infection site and further accelerate host immune responses in order to combat the invading (opportunistic) pathogen [3]. The impact of TLR4 dependent immunopathological sequelae of PA infection is even more intriguing for the following reasons. Firstly, mice are up to 1000 times more resistant to TLR4 ligands such as LPS than humans, and secondly, IL10 gene deficiency renders mice more sensitive to LPS action [39].

## Conclusions

TLR4 dependent signaling of LPS derived from MDR PA differentially mediates both intestinal and splenic immune responses in murine chronic IL10^−/−^ colitis. Future studies need to further unravel the underlying mechanisms of interactions between MDR (opportunistic) pathogens such as PA, the complex intestinal microbiota and host immunity.

## Additional file

**Additional file 1: Figure S1.** Large intestinal histopathological changes in IL10^−/−^ mice lacking TLR4 following peroral association with multi-drug resistant *P. aeruginosa*. Representative photomicrographs of hematoxylin and eosin stained paraffin sections illustrate histopathological changes in large intestines of conventionally colonized IL10 deficient (WT IL10^−/−^) and TLR4 deficient IL10^−/−^ mice (TLR4^−/−^ × IL10^−/−^) at day 14 following peroral challenge with a clinical multi-drug resistant *P. aeruginosa* (PA) strain. Naive (N) mice served as negative controls. Continuous and dotted arrows indicate mucosal and submucosal infiltrates, respectively. The star points towards a dysplastic crypt. Scale bar: 100 μm (100× magnification).

## Abbreviations

CFU: colony forming units
H&E: hematoxylin and eosin
HPF: high power field
ICU: intensive care unit
IFN: interferon
IL: interleukin
MLN: mesenteric lymph nodes
N: naive
NFκB: nuclear factor ‘kappa-light-chain-enhancer’ of activated B-cells
NO: nitric oxide
MDR: multi-drug resistant
PA: *Pseudomonas aeruginosa*
PBS: phosphate buffered saline
p.i.: postinfection
SPF: specific pathogen free
TNF: tumor necrosis factor
TLR: Toll-like receptor
Treg: regulatory T cell
WHO: World Health Organization
