# Supplementary figures and images for: Toll-like receptor-4 differentially mediates intestinal and extra-intestinal immune responses upon multi-drug resistant Pseudomonas aeruginosa association of IL10−/− mice with chronic colitis
Source: Gut Pathog. 2017 Nov 7;9:61. doi: 10.1186/s13099-017-0211-z (PMC5678768; doi:10.1186/s13099-017-0211-z)

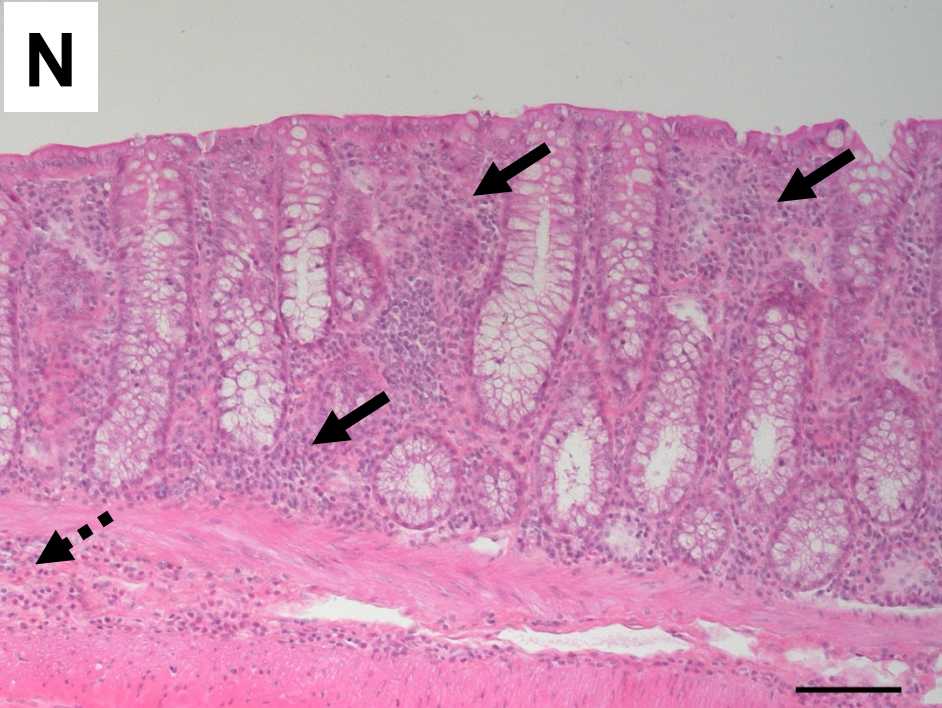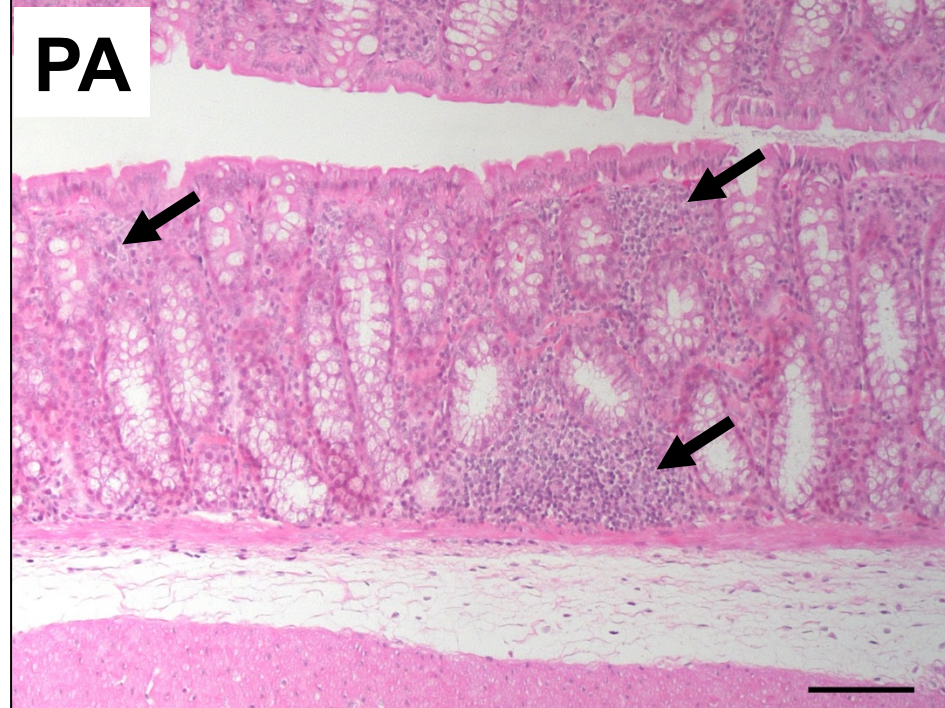

**IL10<sup>-/-</sup>**

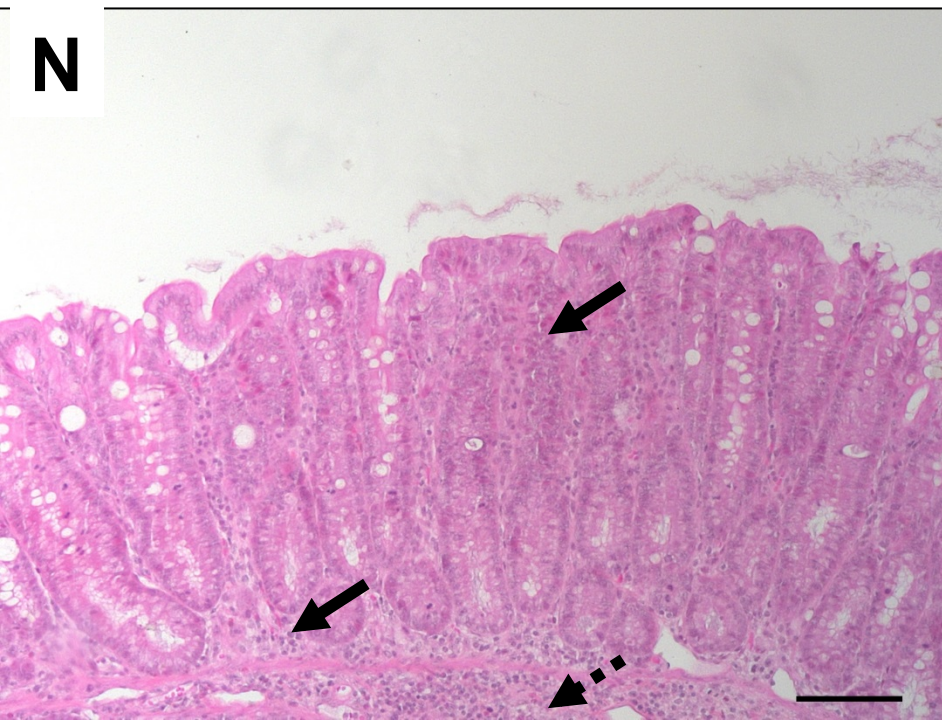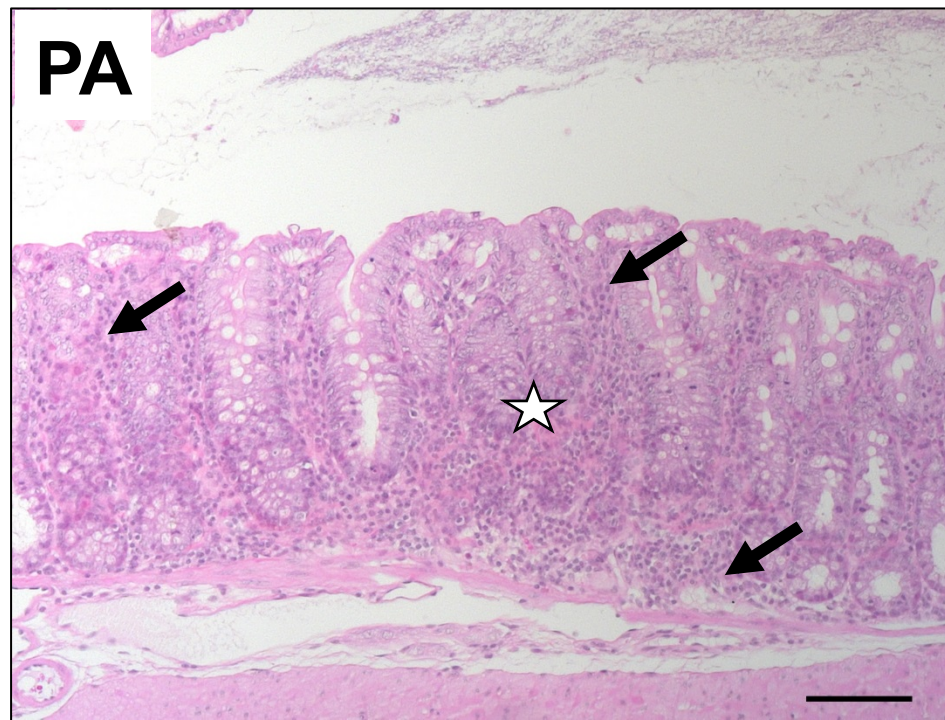

**TLR4<sup>-/-</sup> X IL10<sup>-/-</sup>**

Supplement: Supplementary file 1 — Additional file 1: Figure S1. Large intestinal histopathological changes in IL10−/− mice lacking TLR4 following peroral association with multi-drug resistant P. aeruginosa. Representative photomicrographs of hematoxylin and eosin stained paraffin sections illustrate histopathological changes in large intestines of conventionally colonized IL10 deficient (WT IL10−/−) and TLR4 deficient IL10−/− mice (TLR4−/− × IL10−/−) at day 14 following peroral challenge with a clinical multi-drug resistant P. aeruginosa (PA) strain. Naive (N) mice served as negative controls. Continuous and dotted arrows indicate mucosal and submucosal infiltrates, respectively. The star points towards a dysplastic crypt. Scale bar: 100 μm (100× magnification). [file 13099_2017_211_MOESM1_ESM.pdf]
